# Context Impacts in Accelerometer-Based Walk Detection and Step Counting

**DOI:** 10.3390/s18113604

**Published:** 2018-10-24

**Authors:** Buke Ao, Yongcai Wang, Hongnan Liu, Deying Li, Lei Song, Jianqiang Li

**Affiliations:** 1 School of Information and Communication Engineering, Beijing University of Posts and Telecommunications, Beijing 100876, China; aobuke@hotmail.com; 2 School of Information, Renmin University of China, Beijing 100872, China; deyingli@ruc.edu.cn; 3 School of Software, Tsinghua University, Beijing 100084, China; lhn08@tsinghua.org.cn; 4 Institute for Interdisciplinary Information Sciences, Tsinghua University, Beijing 100084, China; leisong03@gmail.com; 5 School of Software, Beijing Institute of Technology, Beijing 100081, China; lijianqiang@bjut.edu.cn

**Keywords:** walk detection, step counting, gait analysis, machine learning, signal processing

## Abstract

Walk detection (WD) and step counting (SC) have become popular applications in the recent emergence of wearable devices. These devices monitor user states and process data from MEMS-based accelerometers and optional gyroscope sensors. Various algorithms have been proposed for WD and SC, which are generally sensitive to the contexts of applications, i.e., (1) the locations of sensor placement; (2) the sensor orientations; (3) the user’s walking patterns; (4) the preprocessing window sizes; and (5) the sensor sampling rates. A thorough understanding of how these dynamic factors affect the algorithms’ performances is investigated and compared in this paper. In particular, representative WD and SC algorithms are introduced according to their design methodologies. A series of experiments is designed in consideration of different application contexts to form an experimental dataset. Different algorithms are then implemented and evaluated on the dataset. The evaluation results provide a quantitative performance comparison indicating the advantages and weaknesses of different algorithms under different application scenarios, giving valuable guidance for algorithm selection in practical applications.

## 1. Introduction

We have witnessed the rapid growth and wide popularity of smart watches, smart bands and smartphones in recent years. Walk detection (WD) and step counting (SC) are fundamental applications using these smart devices. They also provide the algorithmic basis for daily activity recognition, calorie consumption estimation, body state diagnosis and indoor navigation.

WD and SC are generally carried out by processing sensing data, from an MEMS-based accelerometer and optionally gyroscope sensors. These sensors are generally tiny and low-cost and embedded in the wearable devices. They provide continuous 1D to 3D accelerator or angle rate data, so as to indicate the motion dynamics of the user who is wearing the device. Various algorithms have been proposed to process the accelerator data stream to extract temporal and frequency features from the sensing data and design recognition algorithms for walk detection and step counting. In particular, walk detection could differentiate walking from other daily activities, such as sitting, standing and running. Step counting counts the number of steps when users are in a walking state.

Algorithms were designed using different ways to process the accelerator stream data, in order to conduct walk detection and step counting. The common design methodologies include heuristic-based, signal processing and machine learning methods. However, a common feature of these algorithms is that the design is based on recognizing the patterns of a gait cycle. In particular, a gait cycle of a human is composed of several consecutive phases, i.e., stance phase and swing phase. Algorithms recognize and extract the features of these phases to detect walking, and the distinguished periodical events are exploited for step counting.

However, the algorithms’ performances are shown to be highly sensitive to the application contexts, and the following contexts are used in this paper: (1) the sensor placements; (2) the sensor orientations; (3) the user’s walking patterns; (4) the preprocessing window sizes; and (5) the sensor sampling rates. How the WD and SC algorithms are impacted by the dynamic contexts has not yet been adequately investigated. Most previous works have only treated the sensor placement as the major context factor [1,2,3]. As in Brajdic’s intensive survey [4], they only compared different algorithms and the effects of placements, including [4], who evaluated the performance of step counting algorithms in a loose way. To date, few works have studied the impact of all five application contexts simultaneously within the same experiment. In this paper, we extended the single context factor of placement to five context factors. We also employed a new criterion (Section 5.3.1) to evaluate the performance of step counting.

In addition, existing datasets do not provide enough information in evaluating WD and SC thoroughly. For example, the dataset of [5] is small, and only gestures are collected. USC-HAD [6] fixed the sensor position at the front right hip. The UCI smartphone dataset [7,8,9] only fixed the sensor at limited positions. The PlaceLab dataset [10] only had one subject. The work [11] lacks step data. To evaluate WD and SC thoroughly, we built our own dataset. We consider context factors including placement, orientation, walking pattern factors and also window size and sampling rate. The window size and sampling rate are determined by the potential applications and are also limited by the hardware. We collected and labeled datasets by the five factors.

In the other applications, WD and SC are fundamental algorithms. For example, [12] used SC to assist in indoor navigation; [13] recognized physical activity such as walking, running, etc.; [14] was a real-time gait analysis in walking and running. The work in [15] could detect falls among activities for elderly people. The work in [16] was a low-cost indoor/outdoor navigation system aided by GPS. Many studies have employed multiple sensors such as the accelerometer, gyroscope, heart rate sensor and a barometer to detect human activity. While placing more sensors in different locations can be cumbersome for subjects, one single accelerometer is more preferable due to the low power consumption and low cost.

From the algorithmic aspect, five algorithms from three main categories (heuristic, signal processing and machine learning) are investigated and summarized in this paper. We carefully design experiments to evaluate the impacts of different contexts thoroughly. In each experiment, testing datasets were constructed by recording multiple users’ movement indoors and outdoors, using Android phones and smart watches as the testing devices. The accuracy and fragility of different algorithms provide valuable guidance for algorithm selection for the related applications. Although many commercial wristbands, pedometers and step counting applications have been developed, they usually suffer from false positives. We employ a receiver operating characteristic (ROC) curve to make a more accurate evaluation.

The remaining sections are organized as follows. The related works and context impacts are defined in Section 2. Related algorithms are introduced in Section 3. The experiment design is introduced in Section 4. Performance evaluation and comparisons are presented in Section 5. Section 6 concludes the paper.

## 2. Take Context into Consideration

### 2.1. Context Definition

Because the application contexts are diverse and time varying in real applications, they can hardly be considered in the algorithm design phase. However, they do have observable impacts on the WD and SC performances. To evaluate the impacts of dynamic contexts on the algorithms’ performances, the availability of real-time context knowledge is modeled as variables. In this paper, five context variables are considered, as shown in Table 1.

The knowledge of orientation $I_{O}$ and person information $I_{P}$ are modeled by binary variables. $I_{O}$ indicates whether the sensor orientation is known or not, and $I_{P}$ indicates whether the model is personalized (trained and tested on each subject); the sampling rate *S* and window size *W* are modeled as discrete real numbers, with data range shown in the table; the wearing location is also modeled as a discrete variable, with possible values shown in the table. The available “training” and “testing” data were changed according to the different contexts; for example, if we restrict *L* to being Foot, then only data collected at Foot is available.

Then, let $x=\{x_{1},\cdots ,x_{T}\}$ be the accelerator data sequence collected from 1–*T*, $s=\{s_{1},\cdots ,s_{T}\}$ be the user state ground truth during this period and $n_{T}$ be the step number ground truth. With the consideration of application contexts, $f_{w}\left(x\right)$ is a specific WD algorithm, which outputs the states of whether the user is walking at each time point from 1–*T*. $f_{s}\left(x\right)$ is a specific step counting algorithm, which outputs the estimated number of steps from 1–*T*. Then, the walk detection error with consideration of context impacts is represented by:

(1) $$e_{w,T}=\sum_{t=1:T}\left|s_{t}-f_{w}\left(x,I_{O},I_{P},R,W,L\right))\right|$$

The step counting error is represented by:

(2) $$e_{s,T}=\frac{|n_{T}-f_{s}\left(x,I_{O},I_{P},R,W,L)\right)|}{n_{T}}$$

In the following sections, we introduce walk detection and step counting algorithms, i.e., $\left\{f_{w}\left(x\right),f_{s}\left(x\right)\right\}$, and present the performance evaluations of these algorithms for different application contexts.

### 2.2. Related Works on Context Impacts

Since WD is part of human activity recognition (HAR), we surveyed the HAR works that considered the contexts such as placement and personalization, instead of purely WD. We will also introduce some typical SC works that considered contexts.

(1) Placement is the most common context and is the factor that has attracted researchers’ attention. Olguin et al. placed one or two accelerometers on three different parts of the body and studied the classification accuracy of activity recognition [17]. Lester et al. studied whether a single accelerometer could generalize well on different locations and the reliability of activity recognition on a novel individual [18]. The works in [2,19] explored the influences of placements on different body parts. The work in [1,3] showed that the negative influence of various placements of the sensor could be mitigated. Cleland et al. studied the optimal placement to detect daily activities [20]. Sun et al. investigated the effects of varying positions and orientations on the accuracy of activity recognition [21].
(2) The impact of personalization was investigated in [22]. The work in [22] compared the impersonal model and personal model. The impersonal model was built using the data from many users and tested on a new user; the personal model was a personalized method built with the data from the specified user and tested on him/herself. The result showed that the personalized method was much better than the impersonal model. By using active learning and semi-supervised learning algorithms, [23,24] in fact developed a personalized model based on a original classifier and showed the significant improvement over the original classifier, which was trained on the data from many users.
(3) The impacts of window size were evaluated in [21,25,26]. Although [25] demonstrated that the accuracy was nearly the same under different window sizes, these findings contradicted those of [21,26]. The main reason is that the contexts of the three works were different, which shows the importance of conducting complete evaluations under various contexts.
(4) The impacts of multiple contexts were also investigated in some existing works. The work in [3] investigated the placements, feature selection and the window on/off on the accelerator to evaluate the accuracy of activity recognition. The research showed that the accuracy at the trouser front pocket position had lower accuracy to classify activities and also had difficulty in distinguishing normal walking and fast walking. In addition, the work showed that the classification accuracy between standing and sitting could be significantly enhanced if the sensor position were considered.

Kunze [1,2] evaluated the context impact of placements and orientations. The work considered the placement including head, trousers, torso and wrist. It showed that the displacement of sensors could harm the accuracy, but this could be mitigated by extracting placement-independent features and placement recognition. Besides, it showed that the closely related placements usually generated misclassifications.

For step counting, the Pan-Tompkins method (PTM) [27] only mounted the sensor at the foot and reached a high accuracy in SC. In [28], each subject wore the sensor on his/her waist, and then, the activities were classified and steps counted.

It can be seen that previous activity recognition studies mainly conducted the experiments under a few contexts such as classifiers, placements and orientations of the sensor; therefore, in-depth studies considering complete contexts are needed. Besides, The majority of most past research only studied HAR, which made the evaluations on both WD and SC under various contexts necessary. In our paper, we considered more comprehensive application contexts that include classifiers, placements, orientations, window size, sampling rates and personalization to make a complete comparison.

## 3. WD and SC Algorithms

Walk detection (WD) and step counting (SC) algorithms were generally designed following a similar routine: (1) feature extraction; (2) feature detection; and (3) state recognition. In an implementation, the design methodologies can be roughly categorized into: (1) heuristic-based; (2) signal processing based; and (3) machine learning-based. The related algorithms are briefly summarized and introduced.

Since we aim to give a comprehensive evaluation of various contexts, a proper set of algorithms that could exactly reflect the contexts’ changes should be selected. Some features and algorithms that are too sensitive or not widely applied could not provide fair comparisons of different contexts. Too complicated features and some ‘best’ algorithms should be avoided because they may generate biased results. Therefore, we surveyed many papers on both feature extractions and classification algorithms and selected the most stable features and algorithms to conduct the experiments and evaluations.

### 3.1. Feature Extraction Techniques

Feature extraction from the accelerator data stream is crucial for walk detection and step counting. Various features have been presented in the literature. Statistical features in the time domain, including mean, variance, correlation, skewness, kurtosis, energy, etc., were proposed in [29,30,31,32,33,34]. Other features such as peak interval and zero/mean-crossing rate were also proposed in [9,35]. Additionally, root mean square (RMS) and histogram were proposed in [36].

Features in frequency and transformed domains were also proposed in many works. FFT bins were used in [28,37], and wavelet coefficients were introduced in [38,39]. The peak frequency and power ratio of different frequency bands were exploited in [37]. Mel-frequency cepstral coefficients (MFCCs) and Bark-frequency cepstral coefficients (BFCCs) as complex features of frequency domains were also proposed in [36].

Besides these conventional features, principal component analysis (PCA) was proposed in [40], although it is commonly used as a feature selection method. Autoencoder networks [40] and sparse coding [41,42] have also been introduced recently. Furthermore, some manually designed features such as weightlessness features were used in [43].

We split the common features of WD and SC into three groups in Table 2.

### 3.2. Related Algorithms

#### 3.2.1. Heuristic Methods

Heuristic methods build a series of rules that leverage the cyclic patterns in the time domain to perform walk detection and step counting. The representative algorithms include the multiple threshold method (MT) and the finite state machine (FSM) method. The multiple threshold method, which was proposed by Kim et al. [44], makes use of the cyclic peaks, valleys and thresholds to count steps. The finite state machine (FSM) by Alzantot et al. [35] sets some thresholds in the magnitude to drive an FSM to count steps.

In addition, Randell et al. [45], Bylemans et al. [46] and Ailisto et al. [47] proposed algorithms to detect the step event by finding the consecutive local maxima and minima of the low-pass version of the sensor signal. Beauregard et al. [48] found the positive-going zero-crossing event that indicates the boundaries of each step cycle to count steps. Ying et al. [27] detected the negative peaks that were caused by the heel-strike event to count steps. The correspondence between a peak value and a step was shown in the study of Goyal et al. [49], which finds the peak within one zero-crossing interval when the sensor is placed at the pelvis.

#### 3.2.2. Signal Processing Methods

Signal processing techniques were also exploited to detect walking and to count steps, generally in a transformed domain, by methods such as fast Fourier transform (FFT), short time Fourier transform (STFT) and discrete/continuous wavelet transform (DWT/CWT). Matching methods, such as auto-correlation, cross-correlation, template matching, dynamic time warping (DTW), etc., were also used.

The Pan-Tompkins method (PTM) [27] uses a series of a filter, integration and derivative module to extract step events. STFT [28,50,51] exploits the energy ratio of different frequency bands to perform walk detection, and the period information is used to perform step counting. DWT/CWT [50,52] decompose the original signal into multiple resolutions of the frequency and time domain, which could discriminate walking activity by comparing the ratio between different wavelet coefficients.

Autocorrelation, cross-correlation, template matching and DTW all exploit the similarity between a predefined typical signal of a step cycle and the test sensor data to count steps. The autocorrelation method [53] thresholds the coefficients to detect walk activity and count steps by using the repetitiveness walk activity. Cross-correlation and template matching [27,54] threshold the high positive correlation coefficients to count steps. Ying et al. [27] extracted the first step cycle as the template and computed the normalized cross-correlation to count steps. Although these methods are accurate, the predefined typical template is different in various contexts and hard to find. Similarly, the DTW [55] method measures the similarity between a predefined typical template and the test sensor data, which is time-invariant and robust at various speeds.

#### 3.2.3. Machine Learning Methods

Machine learning techniques such as supervised learning, unsupervised learning, online learning and transfer learning have been investigated for activity recognition and walk detection.

Supervised learning such as decision trees (DT) [56], neural networks (NN), support vector machines (SVM), Gaussian mixture models (GMMs) [32], k-nearest neighbor (KNN) [29], naive Bayes classifiers [28] and boosting methods [57] have been studied and generally achieve good detection accuracy.

Unsupervised methods have been used in activity recognition. For example, hidden Markov models [36,58,59,60] are powerful in sequence data analysis; they could also be used to model the walking activity. K-means clustering [61,62] clusters the data in feature space, where the activities could be identified. Although the recognition accuracy of unsupervised learning is generally lower than supervised learning [36], it exempts people from the costly work of labeling the training data.

Besides these typical learning approaches, Cheng et al. [63] investigated zero-shot learning that could recognize unseen new activities when there were no corresponding samples in the training dataset. Rebetez et al. [64] introduced growing neural gas (GNG) to build an online learning recognition system that did not require labeled data. Transfer learning [65,66] could transfer activity recognition from one domain to another domain, which adapts the changes of sensor position [33,67], activity type [68] or environment scenario [69].

### 3.3. Selected Algorithms for Comparison

We choose some representative algorithms in each category to evaluate the context impacts considering the complexity and practicability. In heuristic methods, threshold (THR) and FSM [35] were selected because they are intuitive and simple to implement in real systems. In the signal processing category, STFT [50], DWT [50] and PTM [27] were selected. We chose STFT and DWT because they are popular and simple to implement. We chose PTM because it has a refined processing chain. In the machine learning category, we chose KNN [29] and SVM as the simple model and complex model, respectively.

**Figure 1:**
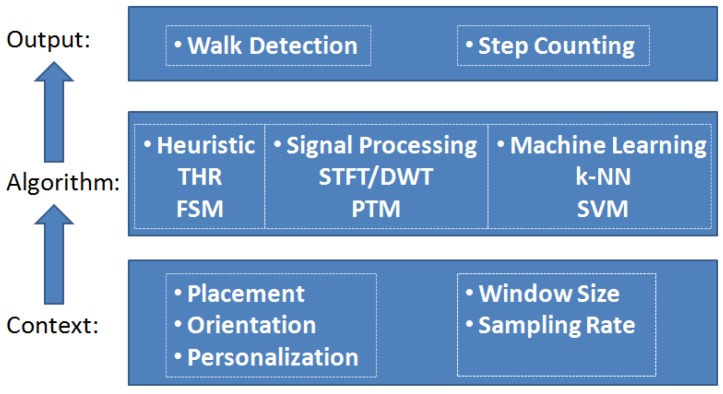
The method of context impacts the algorithm and output. THR, threshold; PTM, Pan-Tompkins method.

These algorithms are chosen from simpler to more complex models considering the requirements and resources of a real system. The evaluation routine is shown in Figure 1.

## 4. Experiment Design

In order to evaluate the contexts completely, we need to design the experiments carefully and correctly. First, we will give a detailed definition of walk and how to distinguish walk from other activities. Then, by simplifying the problem of complex and varying contexts, we could make sure our evaluation is reliable. Last, extensive experiments are conducted, and we will show how to set the parameters of data-preprocessing, feature extraction and model training. We also give an interpretation of false positive in step counting.

### 4.1. Experiment Settings

#### 4.1.1. User Activity Categorization

**Figure 2:**
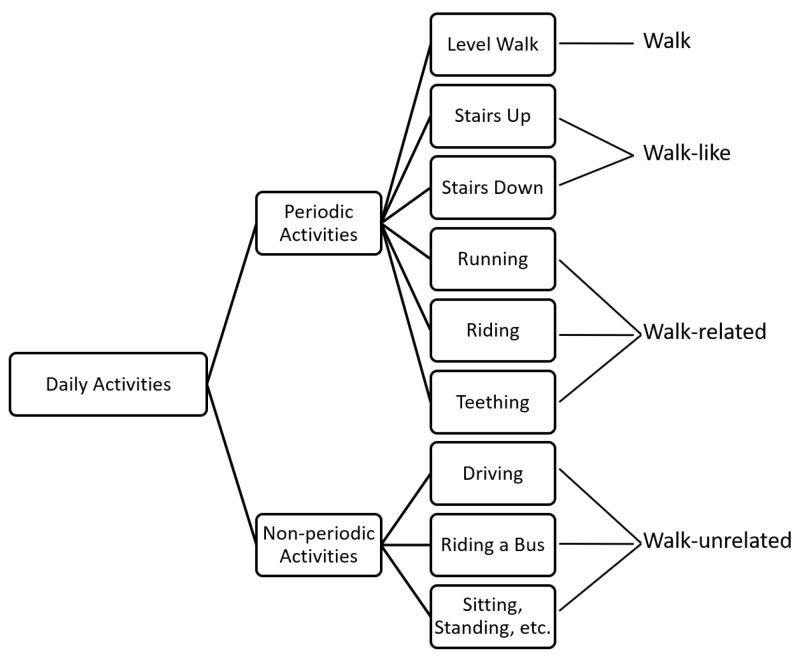
Hierarchical walk activity definition.

Considering the periodic pattern of the walk, we categorized daily activities into two groups: periodic activities and non-periodic activities. Additionally, according to the similarity among these activities, we categorized them into four classes: walk, walk-like activities, walk-related activities and walk-unrelated activities. We show these in Figure 2. Walk-like activities are an extension of walking, which includes going upstairs and downstairs, and could be used in some undemanding systems. Walk-related activities contain those that might be misclassified by algorithms in prior studies, such as running, riding a bicycle and brushing teeth.

In fact, it is quite easy to distinguish walking and walk-like activities from walk-related and walk-unrelated activities. Therefore, we largely simplified the analysis and data collection of walk-related and walk-unrelated in the remainder of the article.

#### 4.1.2. Problem Simplification

In a rigorous way to evaluate the context impacts, we should depict the performance function $f(x_{1},x_{2},\cdots ,x_{i},\cdots ,x_{n})$, 1≤i≤n, where $x_{i}$ is the *i*-th context variable such as placement, orientation, sampling rate, subject, algorithm, age, gender, place of experiment, time of the experiment, sensor type and walking velocity. However, it is impossible to collect data under all these context variables. Instead, we should simplify the experiment by restricting some conditions:

- Sample data at 200 Hz, which is nearly the highest sampling rate of most devices, and downsample it to the evaluation.
- Carry one or two devices at one time and repeat it to cover all the six placements defined in Table 3, since the acceleration data in consecutive rounds within a same building are similar.
- Device orientation of the same placement across different subjects is the same.

In order to evaluate the context impacts of these factors, we should collect data that traverse all the possible cases. Therefore, by exploiting those preliminaries, we conduct our experiments as follows.

#### 4.1.3. Experiment Scheme

**Figure 3:**
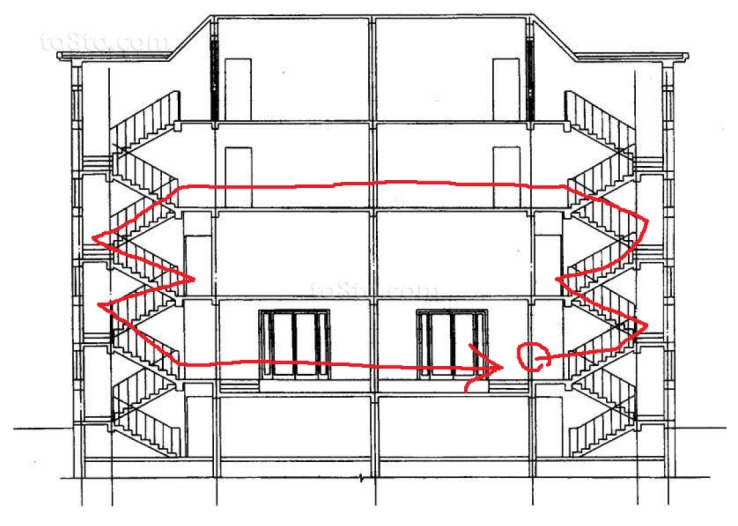
Designed indoor trajectory in data collection.

In order to collect data for our context impact evaluation tasks, it is necessary to have many different kinds of people participating in our experiments. A total of 15 subjects including 10 males and 5 females, with ages ranging from 18 to 28, heights ranging from 1.6 m to 1.85 m and weights ranging from 45 kg to 90 kg, participated in our experiments. Under the assumptions highlighted in Section 4.1.2, we conducted our experiments as follows. Each subject was required to carry multiple smartphones mounted at different positions on the body and walk continuously alongside an indoor track as in Figure 3. This included walking through corridors, going upstairs, going downstairs and going back to the starting point. As we did not use six devices to collect data simultaneously, multiple rounds of walking with the devices mounted at different locations were required to cover all six placements in the data collection. We also employed a camera in this indoor track to obtain the ground truth of step counting. Besides, activities such as running, riding, brushing teeth and driving, as shown in Table 4, do not need to be collected at all six placements, because the signal is similar under different positions. Hand is the subject carries the smartphone in their hand naturally, which is mainly used to simulate the position of the wrist band. Handheld using (HandU) is the subject carries the smartphone, as well as watches the screen, in order to reflect typical walking and using states. The smartphones are not limited to the left or right side; the individual just behaves naturally since we observe that the signal is similar.

Using these assumptions and experiments, we simplified the data collection, and the data could reflect and represent the real contexts well. In the comparison of each context, we only selected data that were generated under the specific context to train the model. For example, we could simply only use the data collected at FrontPocket to train and test the model when we want to get the accuracies of the placements of FrontPocket.

### 4.2. Data Pre-Processing

We calculate the magnitude of a tri-axis accelerometer to remove the orientation constraints in WD and SC tasks except for the evaluation of orientation.

(3) $$d=∥Acc∥=\sqrt{x^{2}+y^{2}+z^{2}}$$

For WD, the raw data of the sensor are first filtered by a low-pass filter with a cut-off frequency of 15 Hz and then segmented into frames of 3 s with 0.5 s overlap. For the evaluation of window size, overlap is always one-sixth of the window size. We record the start time and end time of each activity and then label the data. The activity recognition is performed based on these frames and the labels.

For SC, contexts such as subjects, placements, movement intensities and speeds would cause the deviation of the amplitude, variance, maximum and minimum of the raw sensor data; thus, normalization is necessary. We normalize the raw data by variance since it outperforms other normalization methods such as maximum, minimum and amplitude.

### 4.3. Feature Extraction

Feature extraction is a crucial part of machine learning and has a great influence on the classification performance. Instead of achieving a high accuracy by some complex features in Section 3.1, we aim to evaluate the context impacts and use some popular features in our research. Mean, variation, min, max, energy, skewness, kurtosis, FFT amplitudes, mean-crossing rates and RMS were extracted from the sensor data as features.

### 4.4. Algorithm Parameter Setting

We empirically optimize the parameters of each algorithm and show the implementation details in this section.

THR is used for walk detection. We heuristically threshold the magnitude variance of the sensor data. The optimal threshold value is chosen by exhaustively searching within the range of minimum and maximum variance.

FSM is used for step counting. It has four thresholds that determine the state sequence of the input data stream [35]. We conduct a grid search for the four thresholds used in the model and choose the thresholds that have the best accuracy.

PTM is used for step counting. It includes a series of models such as low-pass filter, differentiator, squaring and integrator, which could find the large sloping part in the sensor data. One local maximum of PTM output means one step. We have the best accuracy when the filter order is 200, the cut-off frequency is 50 Hz and integrator window size is 0.5 s.

STFT is used for both walk detection and step counting. Walk detection by STFT in Barralon et al. [50] places the sensor on the chest, while we do not make this restriction. We first compute FFT coefficients in each frame, then detect walk by setting a threshold on the frequency energy in [0.66 Hz, 1.66 Hz], which outperforms the energy ratio method in [50].

For step counting, we also abandon the placement on the waist, as in [28], and place the sensor at various positions. We add a differentiator module after the energy-based filter that accounts for 20% of the full energy to achieve better performance.

DWT is used for both walk detection and step counting. The decomposition is eight levels by the dh10 wavelet. Instead of comparing the ratio of detail power coefficients, we first smooth the whole energy samples of seven and eight levels’ details of each sample and then detect the walk by considering the mean energy of each window.

For step counting, we reconstruct the signal by 6,7,8 and 2,3 levels’ details, respectively, as two methods, which are denoted as DWT and DWT2, respectively, in the following.

SVM is used for walk detection, and the Gaussian radial basis function (RBF) kernel performs best in our experiments.

k-NN is used for walk detection, and k=5 performs best in our dataset.

Besides the parameters that we mentioned here, there are also many parameters of each algorithm to be decided such as filter order, filter coefficients, differentiator coefficients, etc. In different contexts, we empirically choose these values to achieve the best accuracy.

### 4.5. Counting

Walking is a repetitive activity, which makes the sensor data cyclic. We could see features such as large slope changes, local maxima, local minima, peaks, valleys and mean-crossing events in the magnitude of each gait cycle, where some features could be used to identify and count step cycles. In fact, we expect to detect only one representative event in a gait cycle. However, those features are generated by physical movements, which is intrinsically not stable or uniform distributed in each gait cycle, might emerge more than once in one gait cycle and be sensitive to contexts. For example, more than one large slope change, local maxima, peaks, etc., are observed in one gait cycle because of the context impacts, as well as noises in the physical movement. Therefore, although the algorithm could detect all those features in each gait cycle, step counting is still not accurate since two or more large slope changes might exist in the signal of one gait cycle. In fact, each SC algorithm could detect one feature, for instance each peak in the output of PTM means a large slope change exists in the corresponding position of the input signal. Therefore, simple features, which might emerge more than once in one gait cycle, are inclined to have high false positives; while complex features, which might not appear in a gait cycle, tend to have low true positives. Thus, we need more approaches to balance false positive rate and true positive rate and evaluate the algorithms.

In an algorithmic view, each feature in the gait cycle has one peak in the output, so there might be multiple peaks in the output of each gait cycle. In order to remove the peaks that are generated by noises, we use not only a threshold, but also the minimum peak distance, minimum peak height and minimum peak prominence. These conditions could remove most false positives without the loss of true positives and achieve a better trade off.

## 5. Evaluation Results

We first give a figurative example to present the WD. We apply THR and SVM walk detection algorithms to the continuous activities in a real indoor scenario. The activities consist of a walk, using a phone, going up stairs, going down stairs and some temporal irregular activities such as pushing the door or handshake.

**Figure 4:**
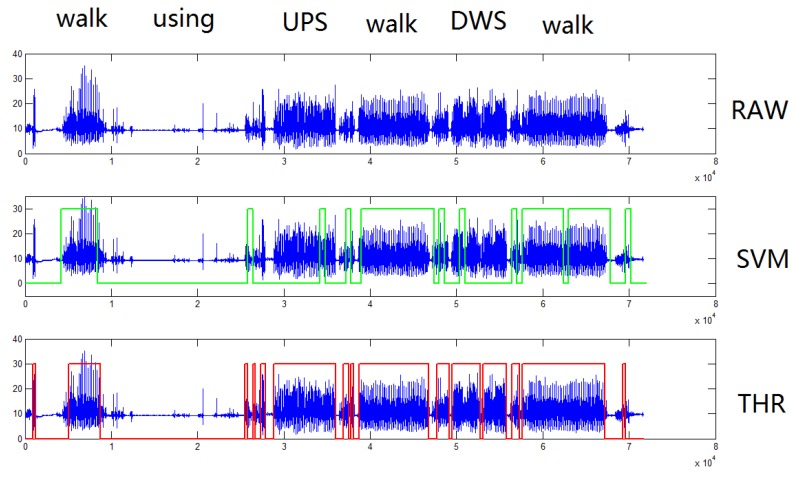
Example of walk detection in a real timeline.

The results are shown in Figure 4. The windows surrounded by red and green rectangles indicate the walking state recognized from SVM and THR, while the low values out of the rectangles are non-walk activities. We saw that SVM provides better classification accuracy than the threshold method in this example. More details will be shown in the following experiments. We could find there are some jitters along the timeline in both algorithms; in addition, the estimated start and end times of walking may deviate from the ground truth.

We have defined four groups: walk, walk-like activity, walk-related activity and walk-unrelated activity. We first address a coarse-grained WD problem that distinguishes walk and walk-like activity from walk-related activity and walk-unrelated activities.

### 5.1. Coarse-Grained WD

Coarse-grained WD is defined to distinguish walk and walk-like activities from walk-related and walk-related activities, which is a coarse classification of walk.

In Table 5, we could find that machine learning methods could easily distinguish walk and walk-like activities from walk-related and walk-unrelated activities. STFT performs better than DWT since walk features are better discriminated in the frequency domain than the time domain.

### 5.2. Context Impacts on Fine-Grained WD

We defined fine-grained WD in order to distinguish a walking activity from a walk-like activity and riding a bicycle. The baseline performance (accuracy) under a predefined context is used to compare with the new performances when more contexts such as placement and orientation are available. Since there are more data on walking than on walk-like activities, we balance the ratio to 1:1 by random selection.

#### 5.2.1. Baseline Performance

The baseline performance of fine-grained WD algorithms are obtained under the sampling rate R=200 Hz and window size W=3 s (600 samples), while orientation $I_{O}$ and personalization $I_{P}$ are unknown and *L* is not specified. All data are trained and tested by 10-fold cross-validation, and the result is shown in the accuracy-base row in Table 6.

#### 5.2.2. Context Effects

We first evaluated the effects of orientation $I_{O}$ and personalization $I_{P}$. Since we could recognize all three axes, if $I_{O}$ is known, then extract features and train all of these on three axes and compare them to the baseline accuracy, which is calculated based only on the magnitude of the sensor data. For the heuristic method and the signal processing method, we chose an axis that performs best on all three axes. Additionally, the accuracies under $I_{P}$ is known are obtained by averaging the accuracy of the test data on each subject. We show the results in Table 6.

If the prior information of orientation $I_{O}$ and personalization $I_{P}$ are independently known, then the performance of WD algorithms is shown in Table 6. If $I_{O}$ is known, then the feature extraction of machine learning algorithms is performed on all three axes, which means that the classifier is trained in the designed orientation. For the heuristic method and signal processing method, we chose the axis that performs best from a gravity axis and a forwarding axis.

We found that both heuristic methods (THR) and signal processing methods (STFT, DWT) are worse than machine learning methods (k-NN, SVM). In fact, heuristic methods and signal processing methods could be viewed as features of machine learning methods. We found that either providing orientation $I_{O}$ or personalization $I_{P}$ could enhance the recognition accuracy, and $I_{P}$ contributed more to the accuracy increasing. If we know the sensor orientation $I_{O}$, there are many techniques to employ them such as extract features in all three dimensions or reconstruct the signal into the Earth coordinate system. Here, we use the common method to extract features in three dimensions of the sensor to observe the impacts. The orientation is different from placement since the sensor could be attached freely without control while the typical placements are fixed, so we could not give comparisons in every orientation. if $I_{P}$ is provided, we only use one’s data to train the personalized model and test the model on him/herself.

**Figure 5:**
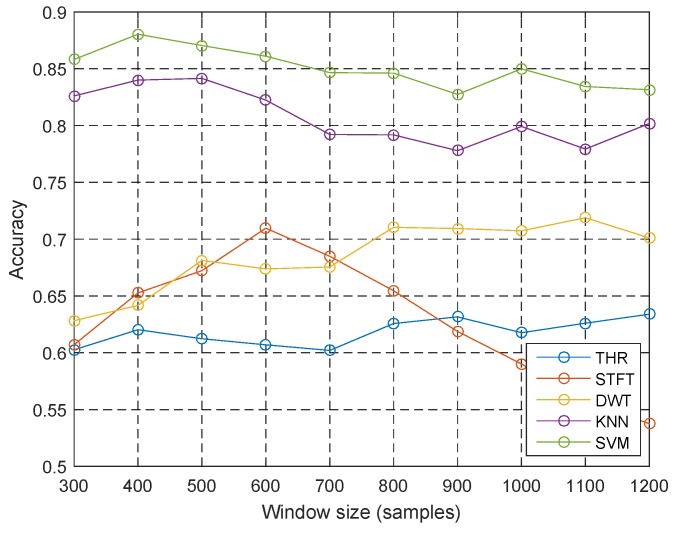
Context effects: window size.

Figure 5 shows the baseline accuracy of algorithms over different window sizes, which varies from 300 to 1200 samples (1.5 s to 6 s). The heuristic methods (THR) are nearly independent of the changing of the window size because the features are nearly time-invariant. The STFT method is best at the window size of 600 samples (3 s). The DWT method becomes slightly higher alongside the increase of window size. Machine learning methods overall become slightly lower along with the increase of window size mainly because the statistical features of different activities in a larger window size are not that easy to discriminate.

**Figure 6:**
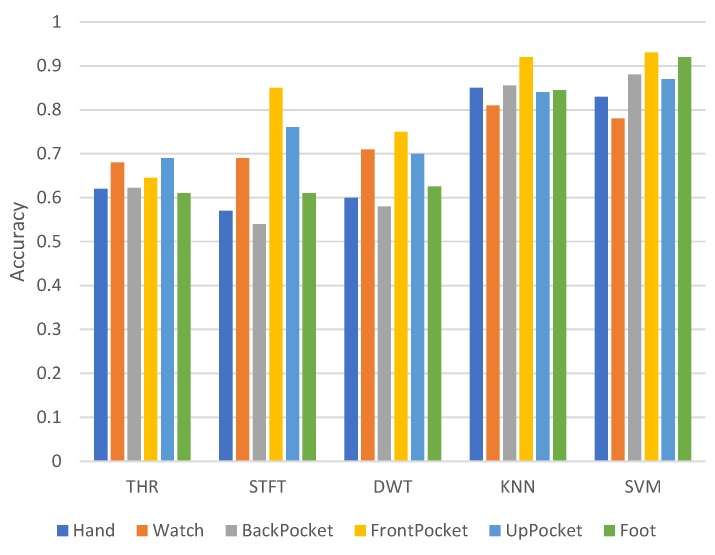
Context effects: placement.

Figure 6 shows the relationship between various placements and accuracy. To evaluate the accuracies of the placements, we selected data only from the specified position to train and test the model and use the accuracies as criteria. The knowledge of placements boosts almost all the accuracies compared to the baseline accuracy, which indicates that placements make the dataset more discriminative. Although some placements slightly increase the accuracy of the heuristic method (THR), it is not sensitive to variations of placements. The STFT method and DWT method perform even worse than the baseline accuracy at some placements. The machine learning methods (k-NN and SVM) display the largest increase in Figure 6. Lastly, the accuracies of STFT, DWT, k-NN and SVM all increased at FrontPocket.

**Figure 7:**
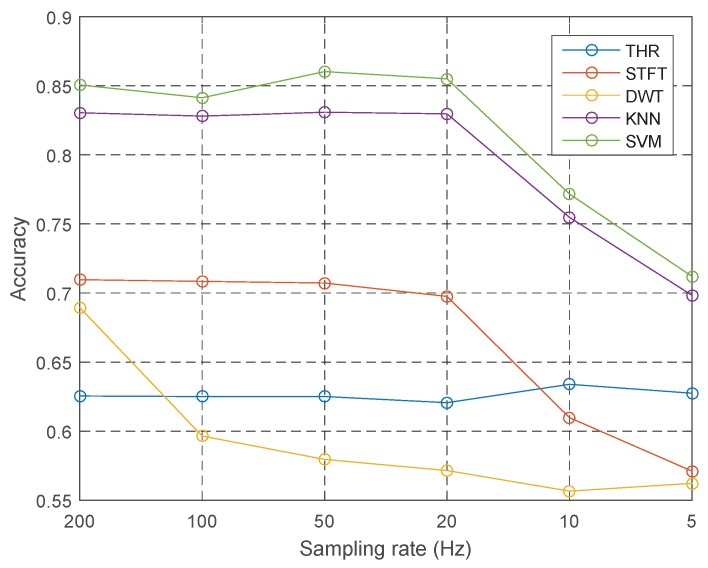
Context effects: sampling rate.

Figure 7 depicts the baseline accuracies under different sampling rates *R*. The accuracy decreases smoothly with the increase of *R*. Furthermore, 20 Hz is the transition point, where the accuracies of k-NN, SVM and STFT diminish quickly. THR is nearly independent of the changes of *R*, since the variance is steady. DWT becomes relatively low when the sampling rate is 100 Hz.

We observed that the machine learning algorithms outperform heuristic and signal processing methods in distinguishing walking activity from other periodic and walk-like activities. This is mainly because the heuristic and signal processing methods suffer from the indistinguishable patterns of variance and spectrum between walking activity and other activities. Besides, the accuracies of heuristic methods and signal processing methods are not noticeably improved even though more contextual information is provided. Besides, the false positives and false negatives of riding a bicycle are much lower than other activities because the sensor movement in the FrontPocket is highly restricted, and false positives are difficult to recognize.

The accuracy of heuristic methods and signal processing methods is a result of a balance among true positives, true negatives, false positives and false negatives. We chose the best accuracy that was larger than 50%, under the condition of both true positives and true negatives.

### 5.3. Context Impacts on SC

The context impacts of SC are performed only on the walking data, without other activities such as going upstairs, going downstairs and riding.

#### 5.3.1. Definition

We propose two definitions to depict the accuracy of step counting algorithms and choose the strict definition in our evaluation.

Loose definition: The most intuitive way to evaluate step counting algorithms is to compare the estimated step counts to real step counts. This accuracy was presented by Brajdic in [4] and similar definitions are in [27,44,51]:

$$\frac{C_{est}-C_{gt}}{C_{gt}}\times 100\%$$

where $C_{est}$ and $C_{gt}$ are the counts of the estimated steps and ground truth steps, respectively. However, this criterion does not consider the false positives during step counting, so it is necessary to introduce a strict definition.

Strict definition: In a walking activity with uniform velocity, we manually mark each gait cycle in the sensor data. Normally, the algorithm could only count one step in one gait cycle, but this may not be true. Suppose that *n* steps are counted within one gait cycle, and if n>0, then there is one true positive step and n−1 false positive steps. Considering this situation, we introduce an ROC curve that includes both the false positive rate and true positive rate to evaluate the step counting algorithms.

(4) $$\mathrm{TPR}=\frac{C_{tp}}{C_{gt}}$$

(5) $$\mathrm{FPR}=\frac{C_{fp}}{C_{gt}}$$

where $C_{tp}$ and $C_{fp}$ are the true positive and false positive counts, respectively; TPR and FPR are the true positive rate and false positive rate, respectively. Note that FPR might be larger than one since $C_{fp}>C_{tp}$ is possible.

**Figure 8:**
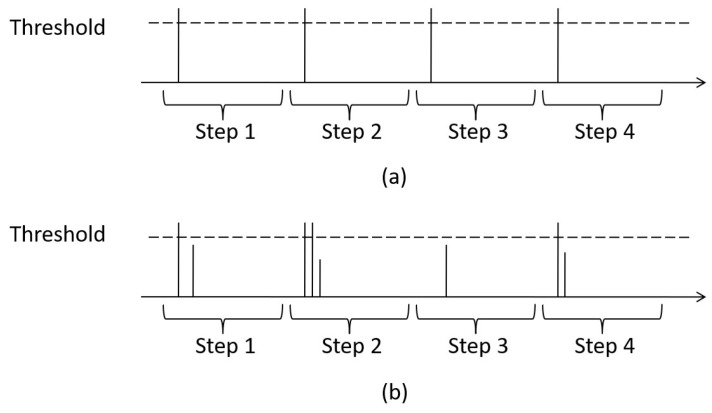
(**a**) Ideal Case of SC Loose definition; (**b**) Bad Case Needs SC Strict definition.

Comparison: All SC algorithms in our paper result in finding the local peaks of a signal, and one step is detected if the peaks are larger than the threshold. Figure 8 shows two examples that have four gait cycles. Compared to the ideal case in Figure 8a that each gait cycle only has one peak, Figure 8b still counts four steps, although one step is lost in the third gait cycle. Thus, our strict definition is more comprehensive to evaluate the step counting algorithms.

We find that the pattern of the step signal has two different groups, which depends on the placements of the sensor. When the sensor is placed at Foot, FrontPocket, BackPocket and Hand, we could observe one period in one gait cycle of one leg. When the sensor is placed at UpPocket or Hand, we could observe two periods in one gait cycle of one leg because the movement of the other leg also has a period. Based on these observations, we separate the placements into two groups: Group I and Group II.

#### 5.3.2. Baseline Performance

**Figure 9:**
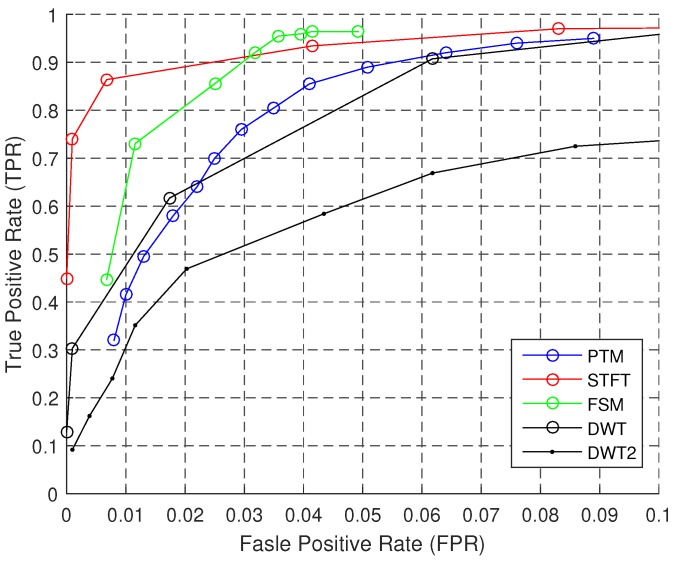
ROC of step counting (Group I).

**Figure 10:**
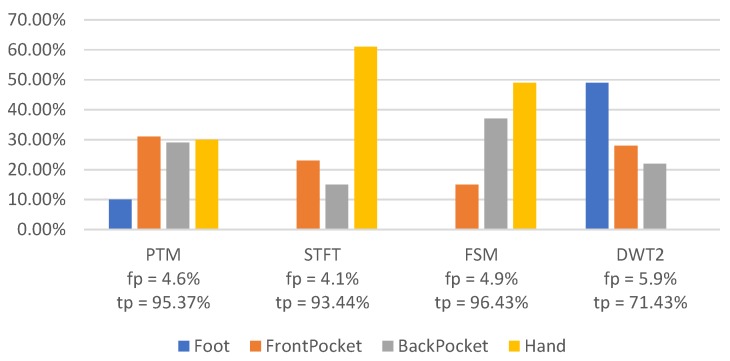
Error sources distribution (fp ≈0.05).

The baseline performance of SC algorithms is obtained under the sampling rate R=200 Hz, orientation $I_{O}$ is unknown, personalization $I_{P}$ is unknown, and *L* is in Group I or II, where Group I contains Foot, FrontPocket, BackPocket and Hand and Group II contains Hand and UpPocket. The baseline performance of Group I is in Figure 9 and Figure 10; the baseline performance of Group II is in Table 7. For Group I, we could observe one period in one gait cycle of the leg that has a sensor; while for Group II, we could observe two periods.

Figure 9 shows the baseline performance of the algorithms under Group I. PTM, STFT and FSM have higher TPR when FPR is 5%. The overall performance of STFT is best, and it keeps improving along with the increase of FPR. FSM has the highest accuracy, while it is not robust, since it performs poorly when the FPR is low; however, this means that one could obtain better accuracy by fine-tuning the parameters. DWT2 performs poor in this case, because the details are not stable features in the signal of a gait cycle. Although PTM includes a series of elegant signal processing modules, the TPR is good only in a short interval (fpr≈4.5%).

Figure 10 reveals the error proportion of each placement that accounts for the total of 5% false positives (approximately). PTM is the most stable algorithm, and most false positives of STFT and FSM happen at Hand.

Table 7 presents the baseline performance of the algorithms under Group II. We could see that these algorithms outperform Group I remarkably, mainly because the features in each gait cycle are consistent under different contexts such as placements and subjects.

#### 5.3.3. Context Effects

When the placement *L* is known, we based the models and corresponding parameters on the data generated by the specified position. Similarly, if the personalization $I_{P}$ is known, then we carried out the same process on the data of each person plus an average process. If the orientation $I_{O}$ is known, we ran the algorithms on all three axes and chose the axis with the best accuracy.

**Figure 11:**
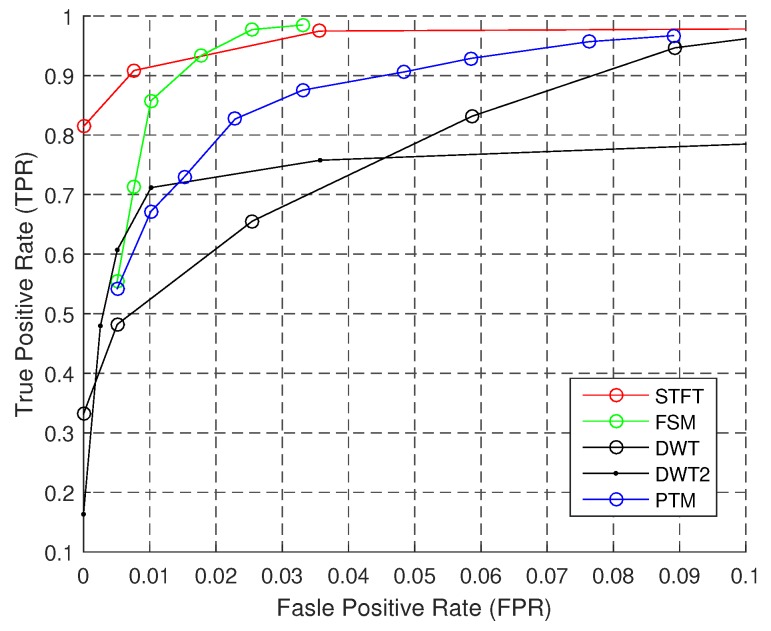
ROC of step counting: personalization.

Figure 11 displays the performances of algorithms when the personalization $I_{P}$ is known. The ROC curve is better than the baseline performance. Compared to the baseline performance, the TPR of FSM and STFT is larger than 95%, and the FPR is lower at the same time. Although the TPR of DWT increases with FPR, the overall performance is poor.

Table 8 shows the TPR and FPR when the sensor is mounted on the foot. In this situation, all algorithms perform excellently except DWT and DWT2 because the detailed component of the sensor data is not remarkable. The overall performance ranking of algorithms is similar to the former experiment: STFT > FSM > PTM > DWT2 > DWT. Sensor data under this circumstance are very regular, which leads to a much better performance.

**Figure 12:**
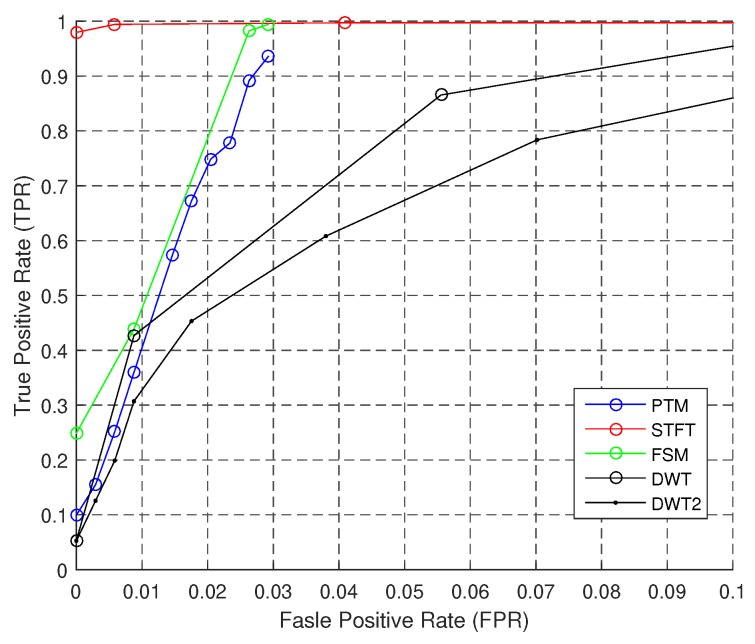
ROC of step counting: FrontPocket.

Figure 12 exhibits the ROC curve when the sensor is in the FrontPocket. The performance ranking is similar: STFT > FSM > PTM > DWT > DWT2. Unlike those former figures, STFT outperforms other algorithms overwhelmingly. FSM and PTM have a high TPR within only a short interval of FPR, which means instability in real applications.

**Figure 13:**
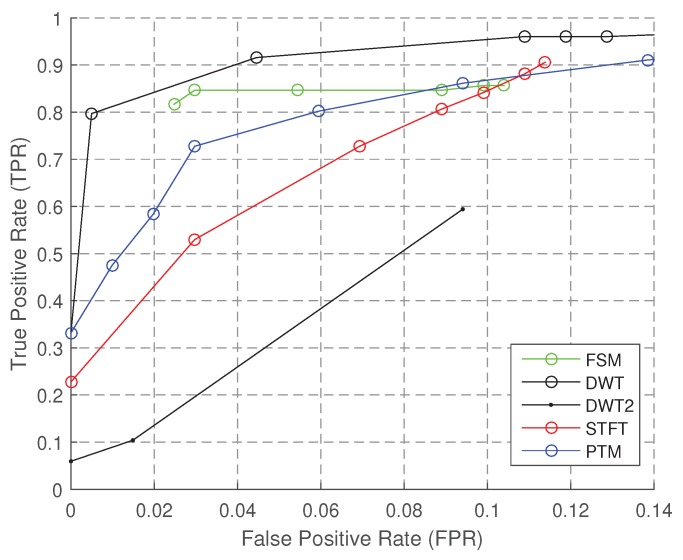
ROC of step counting: Hand.

Figure 13 illustrates the performance of algorithms when the sensor is Hand. DWT outperforms other algorithms, and FSM is more stable than prior placements. STFT does not perform as well as other prior placements. In this case, all algorithms suffer from higher FPR compared to other prior placements, because the hand movement is diverse.

**Figure 14:**
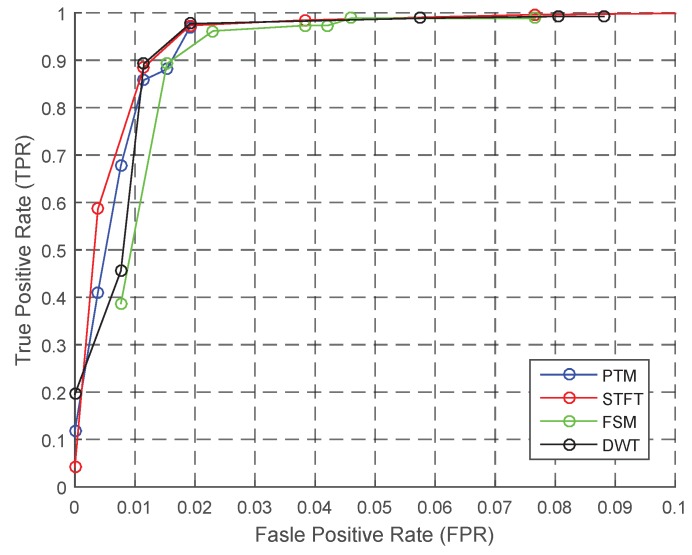
ROC of step counting: BackPocket.

Figure 14 demonstrates the ROC curve when the sensor is in BackPocket. None of the algorithms display great differences under this situation, except that DWT2 performs too poorly to present. We could observe that it is nearly impossible to achieve a high TPR when FPR is low, while the TPR is acceptable at FPR ≈2%. Furthermore, the TPR is almost unchanged, although we allow larger FPR. We could find that all algorithms’ TPR is high at the point of fpr≈2%.

Since it is difficult for people to do anything for a long time when walking, we abandon the evaluation of placement of HandU. Besides the influence of placements, we also investigate the influence of sensor orientation and signal sampling rate and directly gather the results into Table 9 which will be explained in the next section. If orientation is provided, we choose the axis that has the best accuracy.

Table 9 and Table 10 are calculated by comparing the new performances when more contexts are available, in addition to the baseline performances.

### 5.4. Design Rules

We summarize some guidelines to design the WD and SC algorithms regarding the experiments in this research:

- Although machine learning methods perform best overall, STFT could achieve an acceptable level of accuracy when we detect walk and walk-like activity from the other two activities; 20 Hz is the transition point of the sampling rate.
- Among all the contexts, personal info is most contributive where the model is trained on a specific person.
- Complex does not mean accurate: in SC, STFT and FSM perform better in most test cases; PTM is trivial; and DWT overall performs less productive comparatively except at Hand.
- If sensors are mounted on foot, then the noise is minimal, and the result is most reliable.
- Although the steps are accurate, they may suffer from miss counting and false positives; please see the strict definition of SC.

## 6. Conclusions

This paper introduces context factors to evaluate walk detection and step counting algorithms through a series of experiments. Additionally, to the best of our knowledge, the method that uses ROC to evaluate the step counting is new and more comprehensive.

Table 10 shows the context impacts on WD algorithms. We find that different context factors have different effects on the algorithm performance. Amongst all algorithms, heuristic methods (THR) are the most robust to various context changes, while signal processing methods are most sensitive to changes in placement, window size and sampling rate. Machine learning methods have the best performance when a predefined context is given compared to the baseline performance and could be further improved if more contexts are provided.

Table 9 shows the context impacts on SC algorithms. The contribution of orientation is not obvious, while the contribution of personalization could remarkably enhance the overall accuracy except for the DWT algorithm. Besides, all SC algorithms are sensitive to placements, and each placement has its own best algorithm. Finally, when the sampling rate is larger than 20 Hz, the performance of all algorithms remains robust.

This paper seeks to establish a connection between activity recognition and context awareness. By presenting a quantitative comparison of algorithm performance under context impacts, this paper gives valuable guidance in designing algorithms for walk detection and step counting.

## Figures and Tables

**Table 1:** Notations and description of context variables.

Notation	Description	Values
$I_{O}$	Orientation	known, unknown
$I_{P}$	Person info	known, unknown
*R*	Sampling rate	5 Hz–200 Hz
*W*	Window size	1.5 s–6 s
*L*	Wearing location	Jacket pocket (FrontPocket), trouser back pocket (BackPocket), trouser front pocket (UpPocket), foot-mounted (Foot), handheld (Hand), handheld using (HandU).

**Table 2:** Feature categorization of walk detection (WD) and step counting (SC). BFCC, Bark-frequency cepstral coefficients.

Category	Features
Time Domain	mean, variance, peak, peak interval, skewness, kurtosis, energy, entropy, correlation coefficients, RMS, zero/mean crossing rate
Frequency Domain	FFT bins, wavelet coefficients, MFCCs, BFCCs, peak frequency, spectral entropy, power ratio of different frequency bands
Other	PCA, autoencoder networks, sparse coding, weightlessness feature

**Table 3:** The context variables in the experiments.

Context Variable	Settings
Activity	Walk, non-walk (stairs up, stairs down, riding, etc.)
Placement	UpPocket, BackPocket, FrontPocket, Foot, Hand, HandU
Sampling rate	10 Hz, 50 Hz, 100 Hz, 200 Hz
Orientation	Free direction
Individual difference	Age, gender, height, weight, etc.

**Table 4:** Mounted placements of activities in data collection.

Activities	Covered Placements
Level Walk, Stairs Up, Stairs Down	Hand, HandU, BackPocket, FrontPocket, UpPocket, Foot
Running	Hand, BackPocket, FrontPocket, UpPocket, Foot
Riding, Brush Teeth, Driving, Riding bus, Sitting, Standing	FrontPocket, Hand

**Table 5:** Accuracy of coarse-grained walk detection.

	THR	STFT	DWT	k-NN	SVM
Accuracy	77.55%	85.3%	80.7%	96.91%	97.5%

**Table 6:** Context effects: orientation and personalization.

	THR	STFT	DWT	k-NN	SVM
Baseline Accuracy	62.55%	70.96%	67.66%	82.61%	85.57%
Accuracy ($I_{O}$ is known)	62.22%	62.95%	68.91%	92.27%	93.07%
Accuracy ($I_{P}$ is known)	71.03%	83.15%	77.44%	97.62%	96.86%

**Table 7:** Performance of step counting (Group II).

	PTM	STFT	FSM	DWT	DWT2
TPR	91.0%	97.2%	95.7%	98.1%	80.0%
FPR	0.3%	0.52%	0.1%	0.3%	0.3%

**Table 8:** Step counting: Foot.

	PTM	STFT	FSM	DWT	DWT2
TPR	98.7%	99.57%	99.13%	92.61%	95.65%
FPR	1.73%	0%	0%	5.21%	3.47%

**Table 9:** SC accuracy under various contexts (FPR ≈ 3%).

Group	Algorithm	Orientation	Personalization	Placement (Hand, Foot, etc.)	Sampling Rate (5 Hz to 200 Hz)
Heuristic Method	FSM	+1.5%	+9%	[−5%, +10%]	[−32%, +0%]
Signal Processing	PTM	+1%	+16%	[−49%, +17%]	[−28%, +0%]
	STFT	+3%	+5%	[−46%, +9%]	[−25%, +0%]
	DWT	+1%	−1%	[−8%, +28%]	[−30%, +0%]
	DWT2	−11%	+11%	[−42%, +31%]	[−40%, +0%]

**Table 10:** WD accuracy under various contexts.

Group	Algorithm	Orientation	Personalization	Placement (Hand, Foot, etc.)	Window Size (1.5 s to 6 s)	Sampling Rate (5 Hz to 200 Hz)
Heuristic Method	THR	−0.3%	+9%	[−3%, +8%]	[−2%, +5%]	[−1%, +1%]
Signal Processing	STFT	−8%	+13%	[−15%, +15%]	[−16%, +1%]	[−13%, +1%]
	DWT	+1%	+10%	[−8%, +9%]	[−5%, +4%]	[−12%, +1%]
Machine Learning	k-NN	+10%	+15%	[−1%, +10%]	[−6%, +7%]	[−12%, +3%]
	SVM	+8%	+11%	[−8%, +7%]	[−2%, +2%]	[−12%, +2%]
